# BodyThink program-based body image education improves Korean adolescents’ attitudes toward cosmetic surgery: randomized controlled trial

**DOI:** 10.1186/s12912-023-01649-3

**Published:** 2023-12-18

**Authors:** Hyeonhwa Sung, A Young Han, Geun Hee Seol

**Affiliations:** 1 Department of Basic Nursing Science, College of Nursing, Korea University, Seoul, Republic of Korea; 2 Department of Nursing, College of Life Science and Industry, Sunchon National University, Suncheon, Republic of Korea; 3 BK21 FOUR Program of Transdisciplinary Major in Learning Health Systems, Graduate School, Korea University, Seoul, Republic of Korea

**Keywords:** Body image, Depression, Cosmetic surgery, Adolescent

## Abstract

**Background:**

The aims of this study were to modify the widely used BodyThink program to suit the circumstances of Korean schools and determine its effects on body esteem, body image, appearance stress, depression, and attitudes toward cosmetic surgery.

**Methods:**

Participants were 184 third-grade students from two middle schools in Korea, who were randomly assigned to a control or intervention group. Two of the participants dropped out; hence, data from 182 students were analyzed. The control group received the existing curriculum for 4 sessions, and the experimental group was provided with 4 sessions of the revised BodyThink program. Before and after the intervention, all participants completed questionnaires.

**Results:**

In the BodyThink group, improved body image, decreased depression, and positive improvements in attitudes toward cosmetic plastic surgery were observed after the intervention.

**Discussion:**

These results suggest that school health nurses can utilize interventions based on BodyThink program in their curricula to improve the physical and emotional health of adolescents.

**Trial registration:**

This study has been retrospectively registered with the Clinical Research information Service (CRIS) in Korea on October 5, 2023 (KCT0008839).

## Background

Body image formation during adolescence plays an important role in self-perception, self-esteem, and overall quality of life [1]. The transition to adolescence sees children become more aware of their physical appearance, including their height, weight, and facial features, and increasingly compare themselves to social standards and peer groups. Body image is closely related to self-esteem in adolescence. A more positive body image is associated with a higher tendency for positive rational acceptance, whereas a negative body image is associated with a tendency for maladaptive coping, including binge eating [2].

Body image formation in adolescents is greatly influenced by peers and social media. They may compare themselves to friends, celebrities, and influential people, which can affect how they perceive their bodies [3]. Media, including magazines, television, and social media platforms, often portray unrealistic beauty standards. Social media use by adolescents can lead to long-term exposure to such images, which can create perceptions of ideal bodies and cause dissatisfaction with their own bodies [4].

According to the Korean Youth Health Behavior Survey, one in four Korean female students with a body mass index within the normal range had a distorted body image and perceived themselves as overweight [5]. This means that female Korean teenagers show a level of dissatisfaction with their body image that is not in line with their actual appearance. In particular, Korean teenagers are widely exposed to media genres such as K-pop and K-dramas that promote specific beauty standards [6]. In addition to the cultural trend of viewing appearance as a means for social success, Koreans adolescents have long considered cosmetic surgery to be an easy way to improve their appearance [7].

Plastic surgery involves reconstructing or modifying parts of the human body. Reconstructive surgery aims to rebuild or improve the function of a part of the body, such as removal of burns or micro-scars, whereas cosmetic surgery aims to improve the appearance of the body even in the absence of physical or medical problems [8]. Cosmetic surgery has recently become increasingly popular as a means of pursuing beauty by people who are dissatisfied with their appearance [9]. Korea ranks first in the world in terms of cosmetic procedures per capita, with 13.5 procedures per 1,000 people [10], and ranks 5th according to the estimated number of plastic surgeons per 1,000 population. This excessive interest in cosmetic surgery is having a great influence on adolescent students [11].

Negative body image during adolescence can give rise to physical and psychological problems such as eating disorders and bullying, and can continue into adulthood, causing problems throughout life [12]. For this reason, it is essential to guide adolescents toward a healthy body image rather than view their excessive interest in their appearance as a minor problem [13]. To date, educational programs aimed at improving body image have been deployed based on a variety of topics and approaches both domestically and internationally. Among them, the BodyThink program, which was developed by the British Eating Disorders Association with the aim of improving body image and self-esteem and cultivating the ability to critically view images in the mass media, has been shown to improve body image and self-esteem in Australian youth [14]. The purpose of the present study was to apply the BodyThink program to Korean adolescents and determine its effects on body esteem, body image, appearance stress, depression and cosmetic surgery attitude. The results of this study are expected to be helpful in providing effective information on how to promote healthy body image to school and community nurses in charge of the physical and emotional health of adolescents.

## Methods

### Participants

Participants were 184 third-grade students from two middle schools in Gyeonggi-do, South Korea. The study was conducted with the approval of the school principal, and classroom units were randomly assigned to intervention or control groups. In brief, an author of this study, AH, randomly assigned each class to the control or intervention group using a random number table in Microsoft Excel, and notified the researcher HS, who conducted the actual classes, of the results of the assignment. To reduce contamination effects from intergroup communication, classes were conducted without informing participating students whether they were assigned to the control or intervention group. In addition, the purpose of the research and the meaning of participating in the research were explained at each session, and the participants were instructed not to share class content. Participants were eligible for selection if both they and their parents agreed to participate in the study, and if they did not have disabilities in hearing, vision, or literacy. Students who were receiving hospital treatment or counseling for body image-related diseases, students with physical or emotional disabilities, and students who had completed body image promotion education were excluded.

**Fig. 1:**
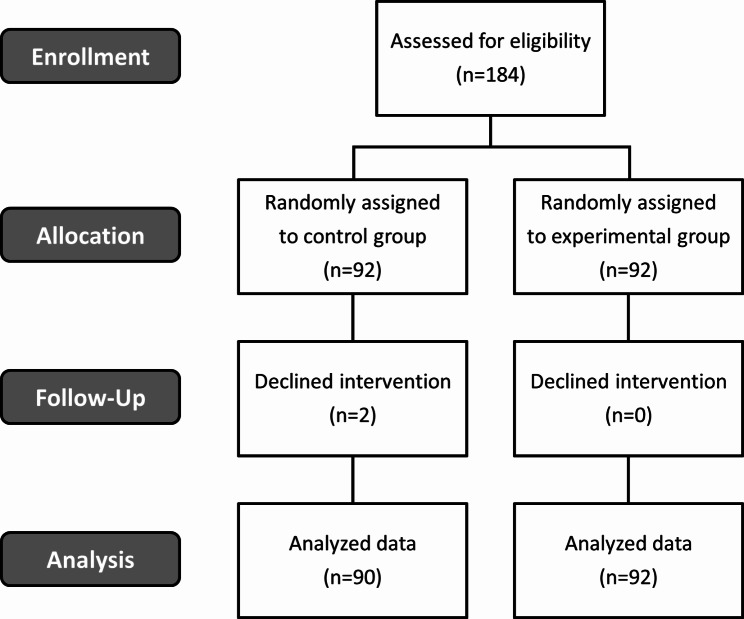
Flow diagram of the study and number of participants at each stage

G*power 3.1 was used to determine the number of subjects needed to achieve a power of 80%, significance level of 0.05, and effect size of 0.4, where these levels were set based on previous research [15]. Assuming a 15% dropout rate, a total of 184 students were selected as the sample size. A total of 92 students were enrolled in the experimental group and 92 in the control group, of which 2 students dropped out in the experimental group. Hence, the final analysis included 90 students in the experimental group and 92 in the control group (Fig. 1).

### Body esteem instruments

Body esteem refers to an individual’s subjective perception and evaluation of his or her own body. Body esteem was assessed using the scale developed by Mendelson, Andrews, Balfour, and Buchoiz (1997) for adolescents and adults, adapted by Lee [16, 17]. This scale consists of 23 questions on a 4-point Likert scale, with a higher score indicating higher body esteem. Questions 4, 7, 9, 11, 13, 17, 18, 19, and 21, which have negative connotations, were reverse calculated. Cronbach’s α coefficient was found to be 0.90 in the present study, similar to the value of 0.92 obtained by Lee (2001).

### Body image instruments

Body image refers to an individual’s satisfaction with the shape and function of each part of his or her body. Body image was assessed using the body cathexis scale developed by Secord and Jourard [18]. This scale consists of 46 questions on a 5-point Likert scale, with higher scores indicating higher satisfaction with one’s body. Cronbach’s α coefficient was found to be 0.95 in the present study, compared to 0.83 in the study of Secord and Jourard (1953).

### Appearance stress instruments

Appearance stress refers to the psychological pressure felt about one’s overall appearance, and was measured using the instrument developed by Yang [19]. A total of 19 questions are measured on a 5-point Likert scale, with higher scores indicating higher appearance stress. Cronbach’s alpha value was 0.95 in the present study and 0.92 in the work of Yang (1993).

### Depression instruments

The Center for Epidemiological Studies-Depression (CES-D) scale Korean version was used to measure depression [20]. The CES-D scale developed by the American Institute of Mental Health (1971) was translated into Korean by Noh (1992) and revised to 26 questions by adding 4 questions to ensure reliability. The Korean version of CES-D uses a 4-point Likert scale to evaluate the frequency of recently experienced depressive symptoms, with higher scores indicating higher levels of depression. Questions 4, 8, 12, and 16 were back-calculated. This tool has been used several times in Korean adolescents. Cronbach’s α coefficient was found to be 0.91 in the present study, and 0.89 in the study by Cho and Kim (1993).

### Cosmetic surgery attitude instruments

Cosmetic surgery attitude, which refers to an individual’s attitudes toward cosmetic surgery, was assessed using the scale developed by Jeon & Lee [21]. This scale consists of 19 questions on a 4-point Likert scale, with higher scores indicating a more active intention toward cosmetic surgery. This tool consists of 5 sub-categories comprising 4 questions about secrecy regarding cosmetic surgery, 4 questions about the value of cosmetic surgery, 5 questions about conformity regarding cosmetic surgery, 4 questions about payment for cosmetic surgery, and 3 questions about risk tolerance for cosmetic surgery. Cronbach’s α in Jeon’s (2002) study was 0.63 for secrecy regarding cosmetic surgery, 0.75 for value of cosmetic surgery, 0.84 for conformity regarding cosmetic surgery, 0.75 for payment for cosmetic surgery, and 0.79 for risk tolerance for cosmetic surgery, and the corresponding values obtained in the present study were 0.86, 0.87, 0.78, 0.86, and 0.90, respectively.

### Revised BodyThink program

The BodyThink program applied in this study maintained the same topics and activities as the previously deployed BodyThink program [14], but several parts were modified to make it applicable to a Korean context (Table 1). For example, the existing program lasted 50 min per session, but in the present study sessions were 45 min, the regular class time in Korean schools. The intervention consisted of 4 sessions, the same as in the existing program. Some video materials were changed to feature Korean culture and Korean people. To increase intervention fidelity in our study, we implemented three strategies. First, the topics and activities of the revised program were verified for content validity through consultation with two nursing professors. Second, a syllabus was created for each session regarding the educational content provided to the control and intervention groups, and the classes were conducted according to the protocol. Third, to increase the internal validity of the study, the author of the present study, a middle school health nurse (HS), delivered the program to all students.

**Table 1:** Intervention programs for the control and experimental groups

Sessions		Control group	Experimental group
1^st^	Aims	• Understand the characteristics of physical development during adolescence • Accepting one’s physical changes positively	• Understand the characteristics of physical development during adolescence • Understand body image and self-esteem and identify influencing factors
	Development	1. Growth surge • Concept and influencing factors of the growth surge during adolescence • Gender differences in the growth surge 2. Secondary sexual characteristics • Explain secondary sexual characteristics according to gender • Think about your own physical changes 3. Forming a positive body image • Efforts to form a positive body image • Attitude guidance to form a positive body image	1. Physical development during adolescence • Concept and influencing factors of the growth surge during adolescence • Expressing changes in one’s secondary sexual characteristics 2. Body image and self-esteem • Explain definition of physical appearance • Explain definition of self-esteem 3. Watch and discuss Real Beauty Sketch • Discuss factors that affect body image and self-esteem • Presentation on ways to improve body image and self-esteem
	Summary	• Summary of the growth surge, secondary growth, sexual maturity, and physical changes	• Summary of the meaning of physical development characteristics, body image, and self-esteem during adolescence
2^nd^	Aims	• Understanding eating disorders caused by body image distortion • Establishing one’s standards for beauty and presenting a desirable body image	• Establishing one’s standards for beauty and presenting a desirable body image • Reduce internalization of ideal media appearances and body comparisons
	Development	1. Body image distortion • Teaching materials: News related to adolescent eating disorders • Learn about the problems of anorexia • Learn about the problems of bulimia • Calculate one’s BMI and think about ways to maintain an appropriate weight 2. Establishing a desirable body image • Setting your own standards for healthy beauty • Identifying ineffective weight management	1. Improve media literacy • Teaching material: Killing Us Softly III video • Explain the definition of media literacy • Discuss the impact of media images on the public after watching the video • Think critically about appearance stereotypes produced by media such as movies, dramas, and advertisements • Establishing your own standards for viewing appearance images produced by the media
	Summary	• Summary of characteristics of eating disorders that may appear during adolescence	• Summary of definition of media literacy and establishment one’s ideal appearance standards
3^rd^	Aims	• Explain how cultural elements such as beliefs, normative practices, and media affect health	• Understand the qualities you value in others • Overcoming negative experiences related to appearance
	Development	1. Health and culture • Explain the concepts of beliefs, practices, norms, and media • Explain the concepts of health beliefs, health practices, health norms, and health media with examples 2. Health promotion culture • Describe a culture that pursues extension of life expectancy and well-being • Describe healthy leisure, healthy company dinners, and healthy foods • Explain that efforts are needed to maintain and promote a health culture	1. Improved self-esteem and recovery from appearance-related injuries • Talk about the effects of teasing one’s appearance • Participate in role-playing about appearance-related teasing • Think about ways to deal with appearance-related teasing • Write and exchange letters complimenting each other on appearance, personality, etc. • Read the letters and share your impressions 2. Understanding others • Sharing opinions on what makes people beautiful
	Summary	• Summary of the impact of culture on health and health promotion culture	• Summary of the effects of appearance-related teasing and countermeasures
4^th^	Aims	• Identify health risk cultures such as fad imitation and suggest improvements	• Understand the reality and risks of youth cosmetic surgery • Reinforce behaviors that positively impact body image and self-esteem
	Development	1. Health risk culture • Explain the concepts of fashion imitation culture, appearance-oriented culture, safety insensitivity, and addiction culture • Explain the seriousness of the culture of indiscriminate imitation of trends and emphasis on appearance due to the influence of consumer culture and media • Explain the health threats of addiction culture and insensitivity to safety • Think about ways to improve health risk culture in daily life	1. The dangers of adolescent cosmetic surgery and the beauty of choosing oneself • Explain the reality and risks of youth cosmetic surgery • Watch and discuss the Choose Beautiful video • Watch and discuss the video of a girl who was teased for having a rare disease, but became a top model • Complete individual activity sheet ‘What can I do to continue to help my body?’
	Summary	• Summarize the impact of health risk culture on health	• Summary of the effects of appearance-related teasing and countermeasures

### Intervention

The intervention was conducted after school for 4 weeks, once a week from May to June 2023. The control group was given four sessions of healthy body image education linked to the existing curriculum. For example, it covered topics such as the characteristics of physical development during adolescence, establishment of a desirable body image, health promotion culture, and health threat culture. The experimental group was provided with 4 sessions of the revised BodyThink program. The interventions for each group are detailed in Table 1. Pre-data were collected immediately before the first training session, and post-data was collected immediately after the fourth training session.

### Ethical considerations

All procedures in this study were conducted with the approval of the Korea University Institutional Review Board (Code: KUIRB-2023-0141-01). Registration for this study as a CRIS clinical trial has been completed (Code: KCT0008839). Considering the characteristics of middle school students, the study was conducted with the consent of the school principal, parents, and the students themselves prior to data collection. A study description and consent form specifying the purpose of the study, research content and procedures, confidentiality of data, disposal after completion of the study, and possibility of cancellation at any time during participation were sent home, and written consent was obtained.

### Statistical analyses

Data were expressed as numbers and percentages or means and standard deviations. The collected data were subjected to a two-sided test at a significance level of 0.05 using SPSS 28.0. After verifying the normality of the data and homogeneity between groups, the independent t-test, Mann-Whitney U test, paired t-test, and Wilcoxon Signed-Rank test were used to compare the differences in the effectiveness of the intervention program between and within the experimental and control groups. The possibility of gender differences was considered, but because the analysis of the results of this study did not show significant differences between genders, the data were analyzed based on the control group and experimental group [22].

## Results

### Participant characteristics

Of the 182 participants, 105 (57.7%) were boys, and the average age was 14.47 years old. The average BMI of the participants was 21.32 kg/m^2^, and 95 (52.2%) were within the normal range. Ninety-four (51.7%) participants preferred sweet tastes. Among all participants, the average scores for the dependent variables were as follows: body esteem, 2.50 ± 0.52 (out of 4.00) points; body image, 3.39 ± 0.76 (out of 5.00) points; appearance stress, 2.28 ± 0.88 (out of 4.93) points; depression, 1.88 ± 0.51 (out of 3.65) points, and cosmetic surgery attitude, 1.55 ± 0.56 (out of 3.16) points. There was no significant difference in the baseline scores of the participants’ general characteristics, body esteem, body image, appearance stress, depression, and cosmetic surgery attitude between the control and experimental groups, ensuring homogeneity (Table 2).

**Table 2:** General characteristics of study participants

Variables	Total (n = 182)	Control (n = 92)	Experimental (n = 90)	t/x^2^	*P*
Gender					
Male	105 (57.7)	53 (57.6)	52 (57.8)	0.001	0.982
Female	77 (42.3)	39 (42.4)	38 (42.2)		
Age (years)	14.47 ± 0.51	14.43 ± 0.52	14.50 ± 0.50	-0.951	0.342
Height (cm)	166.47 ± 8.07	166.62 ± 8.46	166.32 ± 7.70	-0.032	0.974
Weight (kg)	59.52 ± 13.83	59.90 ± 15.16	59.14 ± 12.41	-0.410	0.682
BMI (kg/m^2^)	21.32 ± 3.77	21.35 ± 3.87	21.30 ± 3.70	-0.051	0.960
< 18.5	36 (19.8)	19 (20.7)	17 (18.9)	-0.198	0.843
18.5–22.9	95 (52.2)	47 (51.1)	48 (53.3)		
23-24.9	23 (12.6)	10 (10.9)	13 (14.4)		
> 25	28 (15.4)	16 (17.4)	12 (13.3)		
Siblings					
Only child	37 (20.3)	17 (18.5)	20 (22.2)	0.625	0.533
With siblings	145 (79.7)	75 (81.5)	70 (77.8)		
Preferred flavor					
Salty	42 (23.1)	23 (25.0)	19 (21.1)	-1.403	0.163
Sweet	94 (51.7)	49 (53.3)	45 (50.0)		
Sour	10 (5.5)	6 (6.5)	4 (4.4)		
Spicy	35 (19.2)	14 (15.2)	21 (23.3)		
Bitter	1 (0.6)	0 (0)	1 (1.1)		
Body esteem	2.50 ± 0.52	2.55 ± 0.54	2.45 ± 0.51	-1.270	0.204
Body image	3.39 ± 0.76	3.39 ± 0.75	3.39 ± 0.69	-0.037	0.971
Appearance stress	2.28 ± 0.88	2.20 ± 0.85	2.36 ± 0.90	-1.271	0.204
Depression	1.88 ± 0.51	1.89 ± 0.55	1.86 ± 0.47	-0.092	0.927
Cosmetic surgery attitude	1.55 ± 0.56	1.51 ± 0.54	1.59 ± 0.58	-1.071	0.284
Secrecy regarding cosmetic surgery	1.81 ± 0.85	1.68 ± 0.82	1.94 ± 0.87	-1.988	0.052
Value of cosmetic surgery	1.67 ± 0.76	1.61 ± 0.72	1.72 ± 0.80	-0.847	0.397
Conformity regarding cosmetic surgery	1.53 ± 0.61	1.48 ± 0.56	1.58 ± 0.65	-0.619	0.536
Payment for cosmetic surgery	1.42 ± 0.64	1.43 ± 0.62	1.42 ± 0.67	-0.462	0.644
Risk tolerance for cosmetic surgery	1.31 ± 0.56	1.33 ± 0.59	1.30 ± 0.53	-0.140	0.889

*Note.* Mean ± SD or n (%)

### Effects of intervention on body esteem and body image

The body esteem scores of both the control and experimental groups increased slightly after training, with no statistically significant difference between the two groups. The body image score of the control group changed only slightly, with a score difference of 0.02 ± 0.39 between before and after training, whereas the experimental group’s score increased significantly from 3.39 ± 0.69 before training to 3.55 ± 0.72 after training (*p* < 0.05, Table 3). After training, there was no statistically significant difference in body image between the two groups. These results show that the BodyThink program can help improve adolescents’ body image.

**Table 3:** Outcome variables of BodyThink program of study participants

Variables	Control (n = 92)	Experimental (n = 90)	Z^*^	*P* ^*^
Body esteem				
Pre	2.55 ± 0.54	2.45 ± 0.51		
Post	2.59 ± 0.53	2.49 ± 0.51		
Difference	0.04 ± 0.28	0.04 ± 0.36	-0.276	0.782
*P*^†^	0.059	0.226		
Body image				
Pre	3.39 ± 0.75	3.39 ± 0.69		
Post	3.42 ± 0.84	3.55 ± 0.72		
Difference	0.02 ± 0.39	0.16 ± 0.56	-1.509	0.131
P^**^	0.824	0.028		
Appearance stress				
Pre	2.20 ± 0.85	2.36 ± 0.90		
Post	2.15 ± 0.89	2.29 ± 0.91		
Difference	-0.05 ± 0.44	-0.07 ± 0.55	-0.217	0.828
P^**^	0.455	0.297		
Depression				
Pre	1.89 ± 0.55	1.86 ± 0.47		
Post	1.82 ± 0.50	1.80 ± 0.53		
Difference	-0.07 ± 0.39	-0.05 ± 0.43	-1.011	0.312
P^**^	0.288	0.046		
Cosmetic surgery attitude				
Pre	1.51 ± 0.54	1.59 ± 0.58		
Post	1.54 ± 0.64	1.40 ± 0.52		
Difference	0.03 ± 0.38	-0.19 ± 0.41	-3.851	< 0.001
P^**^	0.830	< 0.001		

*Note.* Mean ± SD, ^*^Mann-Whitney U test, ^**^ Wilcoxon Signed-Rank test, ^†^paired t-test

### Effects of intervention on appearance stress and depression

The appearance stress scores of both the control and experimental groups were slightly lower after training compared to baseline, and there was no statistically significant difference between the two groups. The depression score of the control group decreased slightly from 1.89 ± 0.55 before training to 1.82 ± 0.50 after training, but the difference was not statistically significant. However, the score of the experimental group decreased significantly from 1.86 ± 0.47 to 1.80 ± 0.53 (*p* < 0.05, Table 3). After the intervention, the depression scores of the two groups did not differ significantly. These results show that the BodyThink program has a positive effect on depression in adolescents.

### Effects of the intervention on cosmetic surgery attitude

After the intervention, there was a significant difference in the change in cosmetic surgery attitude scores between the control and experimental groups (0.03 ± 0.38 vs. -0.19 ± 0.41, *p* < 0.001). Compared to baseline, the cosmetic surgery attitude score of the control group did not differ significantly after the intervention, whereas the score of the experimental group after training was significantly lower (1.59 ± 0.58 vs. 1.40 ± 0.52, *p* < 0.001, Table 3).

**Fig. 2:**
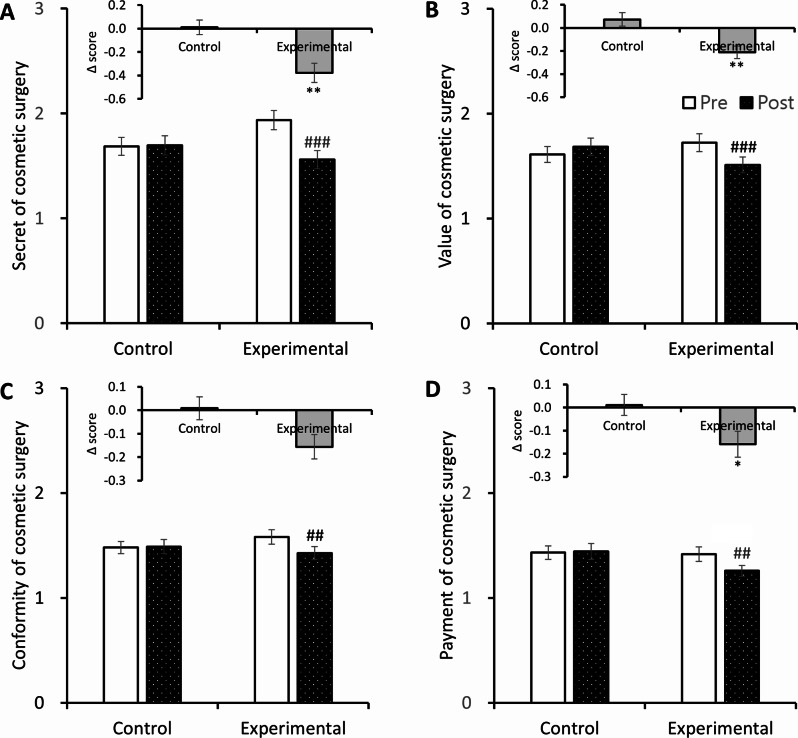
Effect of the BodyThink program on cosmetic surgery attitude subcategories **(A)** Effect of the BodyThink program on secrecy regarding cosmetic surgery. **(B)** Effect of the BodyThink program on value of cosmetic surgery. **(C)** Effect of the BodyThink program on conformity regarding cosmetic surgery. **(D)** Effect of the BodyThink program on payment for cosmetic surgery. Data are expressed as mean ± SEM. ^*^p < 0.01, ^**^p < 0.05 compared with the control group. ^##^*p* < 0.01, ^###^*p* < 0.001 compared with the same group before training

The change in score between before and after the intervention in the cosmetic surgery attitude subcategories differed significantly between the control and experimental groups (Fig. 2). The cosmetic surgery secrecy score, which indicates a person’s desire to hide having undergone cosmetic surgery, of the control group changed very little whereas the score for the experimental group decreased, with the difference between the groups being significant (0.01 ± 0.06 vs. -0.38 ± 0.08, *p* < 0.001). The change in cosmetic surgery value score, which indicates the desire to improve one’s appearance through cosmetic surgery, also decreased significantly in the experimental group compared to the control group (0.07 ± 0.06 vs. -0.21 ± 0.05, *p* < 0.01). The change in cosmetic surgery conformity score, which refers to the psychological pressure to imitate the appearance or opinions of others, showed no difference in the control group, while the experimental group showed a significant change, decreasing by 0.16 from before to after the intervention (*p* < 0.01). The change in cosmetic surgery payment score, which indicates willingness to pay for cosmetic surgery, also significantly decreased in the experimental group compared to the control group (0.01 ± 0.05 vs. -0.16 ± 0.06, *p* < 0.05). These results show that the BodyThink program has a positive effect on the formation of adolescents’ attitudes toward cosmetic surgery.

## Discussion

The purpose of the current study was to evaluate the effects of the revised BodyThink program on overall body image in Korean middle school students. The results showed that the revised BodyThink program can improve body image, reduce depression, and make positive improvements in cosmetic surgery attitudes in Korean adolescents.

Educational interventions to improve body image have been applied on a variety of topics and through a variety of media. Interventions have been based on an ecological systems perspective [23], acceptance and commitment therapy [24], a peer education program [25], emotion regulation [26] and a health belief model [27]. Approaches such as school-based interventions [28], internet-based interventions [29] and application-based interventions are being actively deployed [27, 30]. Systematic reviews have shown that interventions that include topics such as media literacy, cognitive dissonance, and healthy weight information are the most promising approaches for improving body image perceptions in adolescents [31, 32].

In our study, the BodyThink program was found to have positive effects on body image satisfaction. This can be attributed to the emphasis of the BodyThink program on cultivating media literacy to enable adolescents to critically view media images based on their understanding of body image and self-esteem [14]. Given that young people consume large volumes of media and are highly affected by self-esteem issues, the preponderance of content related to media literacy and self-esteem in the BodyThink program makes it particularly suitable for this population [33].

The present results suggest a relationship between improving body image and positive changes in cosmetic surgery attitudes. A previous study showed that young people have a higher level of media involvement than other age groups, and that the higher the level of media involvement, the higher the desire for cosmetic surgery, value attributed to cosmetic surgery, and attitudes toward risk tolerance [34]. Eastern cultures, such as that of Korea, tend to pursue Western face and body shapes as beauty standards, emphasizing certain features such as double eyelids, V-shaped faces, and voluptuous breasts [35]. Adolescents may encounter psychological difficulties such as low self-esteem, appearance stress, and depression if they feel that they must change their appearance to be accepted or valued by their peers or society [4]. Additionally, adolescents are more likely to make decisions about cosmetic plastic surgery without sufficient information about the associated health risks, such as complications or infections [36]. Therefore, education to promote body image can help to free adolescents from social pressures and media portrayals of beauty and help mitigate these physical and mental health risks in adolescents. Depression has been identified as a major predictor of acceptance of cosmetic surgery among Korean women [37]. In the present study, the decrease in depression scores in students who participated in the BodyThink program may be related to the positive change in cosmetic plastic surgery attitudes.

Recently, Korean teenagers have developed a tendency to indulge in sweet foods while imitating ‘mukbang’ influencers on social networks [38]. In our study, we attempted a subgroup analysis based on preferred taste. Although there were no between-group differences according to taste preferences (data not shown), extreme stress regarding appearance often manifests itself as abnormal eating behavior [39]. For example, affected individuals eat a large amount of food in a short period of time and engage in purging behavior or consciously not eating. Therefore, we believe that the relationship between eating behavior and body image is worthy of study.

The students who participated in the present study showed high levels of treatment adherence and low levels of dropout, possibly due to the adjustment of topics and sessions to suit the circumstances of Korean schools. The key to behavior change in education is to change the environment or process to make it easier to adopt a new practice or procedure than a once-and-done strategy [40]. Therefore, providing evidence-based, multifaceted interventions in the school curriculum will be an effective educational method to improve the physical and psychological health of adolescents [41].

This study had several limitations. First, because the intervention program was applied in two middle schools in Korea, research on more diverse institutions and ages is needed to increase the external validity of the educational effect. Second, because one researcher conducted all of the training, it is necessary to verify the program’s acceptability to existing school nurses and school institutions. Third, the present study measured the results immediately after the intervention; hence, there is a need to measure the improvement effects of key variables and whether they are maintained over a long period of time through longitudinal research.

## Conclusion

Body dissatisfaction is a growing public health problem among Korean adolescents. In this study, we confirmed that the BodyThink program, which was modified to suit the circumstances of Korean schools, has a positive effect on adolescents’ attitudes toward cosmetic surgery. Middle school is a critical time in which adolescents form attitudes and behaviors related to body image. Positive interventions at this stage can have a long-term impact on how individuals perceive themselves and others, potentially promoting a healthy body image and reducing appearance-related stress throughout their lives. Therefore, the present results suggest that the BodyThink program should be expanded or integrated into school curricula to benefit greater numbers of students. Additionally, the BodyThink program has the potential to be applied to other rehabilitation populations who experience body image changes during the course of a chronic illness.

## Data Availability

Data are usable on request from the corresponding author on reasonable request.

## Abbreviations

CES-D: Center for Epidemiological Studies-Depression
